# Neonates with Maternal Colonization of Carbapenemase-Producing, Carbapenem-Resistant Enterobacteriaceae: A Mini-Review and a Suggested Guide for Preventing Neonatal Infection

**DOI:** 10.3390/children8050399

**Published:** 2021-05-15

**Authors:** Judy Seesahai, Paige Terrien Church, Elizabeth Asztalos, Melanee Eng-Chong, Jo Arbus, Rudaina Banihani

**Affiliations:** 1 Department of Newborn and Developmental Paediatrics, Sunnybrook Health Sciences Centre, Toronto, ON M4N 3M5, Canada; judy.seesahai@sunnybrook.ca (J.S.); paige.church@sunnybrook.ca (P.T.C.); elizabeth.asztalos@sunnybrook.ca (E.A.); Jo.Arbus@sunnybrook.ca (J.A.); 2 Infection Prevention and Control, Sunnybrook Health Sciences Centre, Toronto, ON M4N 3M5, Canada; Melanee.Eng-Chong@sunnybrook.ca; 3 Neonatal Intensive Care Unit Pharmacy, Sunnybrook Health Sciences Centre, Toronto, ON M4N 3M5, Canada

**Keywords:** carbapenem-resistant Enterobacteriaceae, carbapenemase-producing Enterobacteriaceae, New Delhi metallo-ß-lactamase, neonates

## Abstract

Carbapenemase-producing, carbapenem-resistant Enterobacteriaceae (CP-CRE) are highly drug-resistant Gram-negative bacteria. They include New Delhi metallo-ß-lactamase (NDM)-producing carbapenemase (50.4% of all species in Ontario). Antibiotic challenges for resistant bacteria in neonates pose challenges of unknown dosing and side effects. We report two antenatally diagnosed CP-CRE colonization scenarios with the NDM 1 gene. The case involves extreme preterm twins who had worsening respiratory distress at birth requiring ventilator support, with the first twin also having cardiovascular instability. They were screened for CP-CRE, and a polymyxin antibiotic commenced. In the delivery room, neonatal intensive care unit (NICU) and the follow-up clinic, in collaboration with the interdisciplinary group, contact precautions and isolation procedures were instituted. None of the infants exhibited infection with CP-CRE. Consolidating knowledge with regard to CP-CRE and modifying human behavior associated with its spread can mitigate potential negative consequences. This relates to now and later, when travel and prolific human to human contact resumes, from endemic countries, after the current COVID-19 pandemic. Standardized efforts to curb the acquisition of this infection would be judicious given the challenges of treatment and continued emerging antibiotic resistance. Simple infection control measures involving contact precautions, staff education and parental cohorting can be useful and cost-effective in preventing transmission. Attention to NICU specific measures, including screening of at-risk mothers (invitro fertilization conception) and their probands, careful handling of breastmilk, judicious antibiotic choice and duration of treatment, is warranted. What does this study add? CP-CRE is a nosocomial infection with increasing incidence globally, and a serious threat to public health, making it likely that these cases will present with greater frequency to the NICU team. Only a few similar cases have been reported in the neonatal literature. Current published guidelines provide a framework for general hospital management. Still, they are not specific to the NICU experience and the need to manage the parents’ exposure and the infants. This article provides a holistic framework for managing confirmed or suspected cases of CP-CRE from the antenatal care through the NICU and into the follow-up clinic targeted at preventing or containing the spread of CP-CRE.

## 1. Introduction

Congenital and hospital-acquired neonatal Gram-negative bacterial infection is a well-known, significant risk for both term and preterm neonates [1]. Antibiotics, while a lifeline for many neonates, have also presented the challenge of increasing multidrug-resistant (MDR) bacteria amongst this group. The etiology of this resistance and rapid spread on the microscopic level involves the acquisition of resistant genes from other bacteria through mutation and selection [2]. On the macroscopic level, the causes are intricate and heterogeneous, involving human behavior [3]. MDR often means that neonates may be treated inadequately initially and run the risk of medical deterioration with poorer outcomes [4].

### 1.1. Definition of Carbapenem-Resistant Enterobacteriaceae and Terminology

Carbapenem-hydrolyzing beta-lactamases that confer resistance to a broad spectrum of beta-lactam substrates, including carbapenems, are called carbapenemases. Carbapenem resistance can alternatively be conferred through the presence of porin mutations [5].

Carbapenem-resistant Enterobacteriaceae (CRE) are Enterobacteriaceae that are non-susceptible to a carbapenem [6]. They are a group of highly drug-resistant Gram-negative bacteria that include *Escherichia coli* and *Klebsiella* [6,7,8]. In 2019 the Centres for Disease control updated their 2012 CRE Toolkit, detailing the definition of CRE as: Resistance “to any carbapenem antimicrobial (i.e., minimum inhibitory concentrations of ≥ 4 mcg/mL for doripenem, meropenem, or imipenem OR ≥ 2 mcg/mL for ertapenem) or documented to produce carbapenemase” [6]. Public Health Ontario, also in 2019, described carbapenemase-producing Enterobacteriaceae (CPE) as “Enterobacteriaceae that produce enzymes (i.e., carbapenemases) that inactivate carbapenems and several other classes of antibiotics” [9]. Carbapenemase-producing, carbapenem-resistant Enterobacteriaceae (CP-CRE) are those Enterobacteriaceae that are resistant to carbapenem through the production of carbapenemases.

### 1.2. The Scope of the Problem Globally

**Figure 1:**
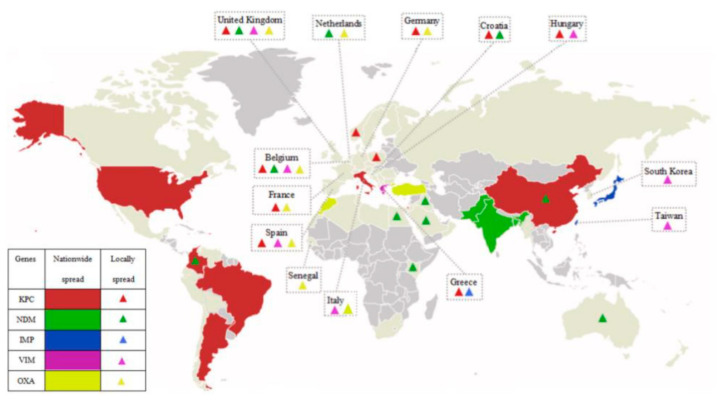
Global distribution of carbapenem-resistant Enterobacteriaceae (CRE). Adapted from Cui et al. [10].

The prevalence of CP-CRE worldwide has been increasing, and concomitantly the challenge of treatment and prevention of dissemination presents itself [6]. In their 2019 article, Cui et al. provided an overview of the global distribution of carbapenem-resistant Enterobacteriaceae (CRE) (see Figure 1 below, adapted from their article) [10].

Different strains predominate in different geographical areas, as is illustrated by the map, first appearing at different times in different regions [10]. The mid-2000s saw the emergence of multiple carbapenemases in the same isolates of Enterobacteriaceae. The Centers for Disease Control and Prevention (CDC) described CRE as “nightmare bacteria” [11].

### 1.3. The Scope of the Problem in Canada

**Figure 2:**
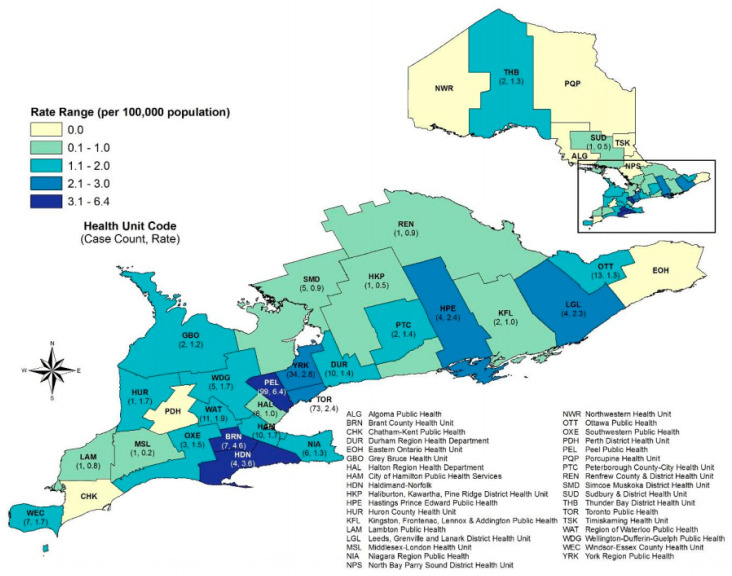
CPE cases and rates by public health unit in Ontario, 1 May 2018 to 30 April 2019 (*n* = 315). Adapted from Public Health Ontario [14].

In Canada, over a five-year period, 261 isolates were reported from participating acute care facilities over a five-year period in 2010 to 2014 [12]. Public Health Ontario has been tracking the rising incidence of colonization and infection of CPE through a mandatory reporting system from May 2018 (Figure 2) [13]. In their November 2019 report, over a period of one year, 315 cases were reported [14].

**Figure 3:**
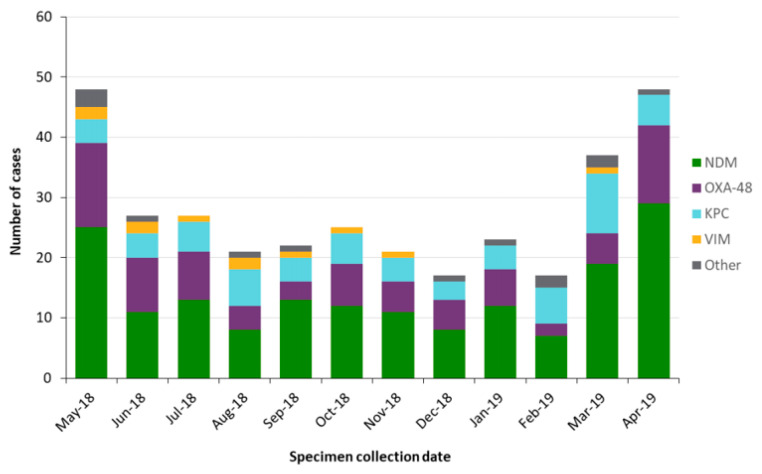
CPE cases by carbapenemase in Ontario, 1 May 2018 to 30 April 2019 (*n* = 333).

They reported that the most frequent isolates that accounted for 70.5% of infections were *E. coli* (50.5%) and *Klebsiella pneumoniae* (20.0%). New Delhi metallo-ß-lactamase (NDM)-producing carbapenemase was the most frequent species, responsible for 50.4% of all species in Ontario [14]. The New Delhi metallo-ß-lactamase-1 gene (NDM-1) was first isolated in 2008 (Figure 3). Since Public Health Ontario instituted mandatory reporting, a variety of strains have been identified and persist as shown in Figure 3.

### 1.4. Risk Factors for CPE and Implications for Neonates Admitted to the Intensive Care Unit

Public Health Ontario identified health care and travel outside of Canada and a medical or surgical procedure in Canada in the last 12 months as risk factors for CPE spread. These are significant risk factors given the demographics of Ontario (Table 1) [14]. This suggests that obstetric cases with medical procedures such as in vitro fertilization (IVF) or IVF patients who received healthcare outside of Canada would likely warrant an assessment for the risk of CPE colonization.

The presence of CP-CRE in the environment in hospital settings, such as in contaminated drains and hand sinks, can act as a reservoir for person-to-person transmission [15,16]. A 2018 Canadian article also supported the suggestion of nosocomial CP-CRE transmission in Canada [17]. Contact precautions, therefore, play a pivotal role [9,18].

### 1.5. Clinical Disease

Asymptomatic colonization with CP-CRE can cause clinical infection. Colonization is mainly of the lower gastrointestinal tract. Infections occur in a variety of systems including the blood, genitourinary tract and lung [9,14,15,19].

## 2. Review of the Medical Literature of CPE in Neonates

A look at the published literature on CRE in neonates, especially with regards to CP-CRE, proves that this is a concern with an international reach (Table 2). Many neonatal studies have looked at the incidence in hospital settings, and all have called for guidelines for management. No published guideline exists for Canadian NICUs.

Travel restrictions due to the current COVID-19 pandemic present a unique opportunity to reset the microbiology clock. We can consolidate our knowledge regarding CP-CRE colonization (usually in the lower gastrointestinal tract) and infection, preparing, therefore, to better modify the human behavior associated with CP-CRE spread and mitigate some of the potential negative consequences when travel and prolific human-to-human contact resumes.

Evidence-based guidelines for CP-CRE exist in different countries, including England [27,28], France [29], the United States of America [6], Australia [30] and Canada [31]. They provided generalized guidelines to minimize spread in healthcare facilities, as this is the main area where the spread is thought to occur. Proactive management strategies and healthcare bundles have been used to prevent spread in the pediatric population [32]. In any maternity hospital, proper adherence to general guidelines for nosocomial infection significantly decreases the CP-CRE infections together with other types of colonization. The guidelines did not, however, specifically relate this to the care of the pregnant mother or the neonate and the setting of the NICU.

### Aim

We aim to provide a framework that can be implemented or modified to local situations for surveillance and prevention of CP-CRE in a maternally colonized high-risk pregnancy and the NICU.

## 3. A Glimpse into Our Local Experience

We present a case from our hospital to illustrate the development of a suggested guideline for management. The infants were twins at 24 + 4 gestational age, born to a 42-year-old mother, G5, A4 (two ectopic and two spontaneous losses) admitted to a level 3 hospital in Canada. 

The mother had in vitro fertilization in India and initially conceived triplets. The pregnancy was reduced to dichorionic diamniotic twins. At 14 weeks gestational age, while still in India, a cervical cerclage was placed. The anatomy scan carried out in India was unremarkable. This mother was known to be carbapenem-producing *Escherichia coli* (*E. coli*)-positive on a rectal swab administered in India. She moved to Canada and had regular visits for her pregnancy. She was noted to have gestational diabetes controlled with diet. Her cervical shortening was monitored. The growth of the twins was appropriate, and the routine serology testing was negative. At 23 + 4 weeks gestational age, she was admitted for progression of cervical shortening. She was transferred to a level 3 hospital due to the risk of preterm delivery. There was no evidence of chorioamnionitis, and no antibiotics were prescribed. This mother received two doses of betamethasone six days prior to delivery. 

Her rectal swab in the level 3 hospital in Canada at 23 + 4 GA demonstrated *E. coli* New Delhi metallo-ß-lactamase (NDM) 1 gene detected by molecular method. It was susceptible to tigecycline with a minimum inhibitory concentration (MIC) = 0.38 mg/L. It was resistant to ceftolozane/tazobactam combination with MIC > 256 mg/L, aztreonam with MIC also > 256 mg/L. Colistin had an MIC <= 0.25 mg/L. It was noted from the lab that there was no clinical data to assist in the interpretation of MIC results for CPE. There is also no Clinical and Laboratory Standards Institute (CLSI) standard testing protocol for the organism and the drug combination. In the 24th week of pregnancy, fetal decelerations were detected in twin A, and the decision was made for emergency delivery by Caesarean section.

The first twin had a spontaneous rupture of membranes two hours prior to delivery. The infant was an extremely low-birth-weight (647 g) male and apneic at birth. Positive-pressure ventilation was commenced due to continued apnea, and he required up to 100% oxygen for recovery from initial bradycardia with a heart rate between 60 and 100 beats per minute. Spontaneous respirations began at three minutes of life, and the infant was placed on continuous positive airway pressure and weaned to room air by about four hours of life. The APGAR score was 3 at 1 min and 8 at 5 min of life. The baby subsequently had recurrent apnea, increasing oxygen requirement requiring intubation and surfactant administration, hypotension requiring fluid boluses, pressor support and a packed red blood cell transfusion. Due to the ill nature of the neonate, empiric antibiotics was commenced with ampicillin and gentamicin. With the further clinical deterioration of potential septic shock, colistin was commenced as per a predetermined plan by an interdisciplinary group in view of mother’s CP-CRE status.

Twin B was born with minimal respiratory effort with a birth weight of 745 g. He required positive-pressure ventilation with a maximum oxygen requirement of 100%. He had an initial good response but then had increasing oxygen requirements and was intubated and surfactant administered. APGAR scores were 7 at 1 min and then 9 at 5 min. He subsequently required ongoing respiratory support but had cardiovascular stability.

Both twins had rectal, endotracheal and axillary skin swabs at birth, in view of the risk of the neonate for resistant *E. coli* colonization/infection. No CP-CRE isolates were identified in the swabs or blood cultures and antibiotics were discontinued.

On day two and day seven of life, CP-CRE screening swabs were carried out. These consisted of endotracheal tube aspirates, rectal, axillary and umbilical swabs. These were all negative for CP-CRE. Please see Table S1 (Supplementary Materials) for a summary of the cultures, results and antibiotic management throughout the admission. During their hospitalization, as expected for extreme preterm neonates, there were instances in which infection was suspected. For these episodes, in addition to the blood, urine or cerebrospinal fluid cultures, swabs were also taken for CP-CRE (as shown in Table S1). They were discharged on day 129 of life at 42 + 6 weeks gestational age.

Throughout the admission, and in collaboration with the interdisciplinary group, contact precautions and isolation procedures were instituted from the admission in the delivery room and maintained on the (NICU). For the medical providers, these included the use of personal protective equipment (gown, mask and gloves). In the resuscitation room, the babies were isolated from the other babies being cared for by providing physical barriers between the patients (curtains). Resuscitation team members were preassigned to ensure no cross-contact with other babies. In the NICU, these measures were continued. Additional precautions were taken with the neonates’ body fluids (stool, urine, secretions in suction catheters) which were contained in tied bags within the neonates’ room until ready for special pick up. The parents were assigned separate inpatient rooms while the mother was admitted with an assigned separate bathroom facility from the general population. This dedicated washroom prevented potential cross-contamination from back splashing and pooled water [15,16]. For the infant care in the follow-up clinic, visits were planned for near the end of the workday, contact precautions were used, and in-depth cleaning of the used room was carried out prior to use the next day.

There has been no need for testing these infants after discharge for CP-CRE as they have not had any signs of sepsis or infection. 

## 4. Discussion

This case report reflects the care practices initiated to contain CP-CRE in a Canadian hospital. CP-CRE are the latest “super bugs,” and treatment options are increasingly limited. Carbapenems were one of the last lines of defense to treat multidrug-resistant Gram-negative organisms, and suddenly even these are being inactivated and hydrolyzed by NDM-1-producing organisms. Many CP-CRE are also resistant to aminoglycosides and fluoroquinolones. Extensive susceptibility testing is necessary for the identification of treatment options, including drugs not usually used or even trialed in the neonatal population, such as tigecycline, fosfomycin, colistin (polymyxin) and rifampin.

There has been a scarcity of antibiotic development in the past 20 years and no new miracle drugs appear to be on the horizon. The medical world is reverting to using medications from the mid-1900s that have since fallen out of favor due to undesirable adverse effects. They appear to be all we have left as a line or defense. Therapeutic options are restricted, and there appears to be no superior treatment regimen. Currently, we resort to individualized management dependent on sensitivities and drug availability.

In this case, an additional restriction was the need for a medication that was licensed by Health Canada (Canada has fewer agents available than the United States or the United Kingdom) and also an antibiotic that could be safely used in an extremely low-birth-weight infant. Our greatest weapon against CP-CRE is therefore prevention.

Active antimicrobial stewardship programs ensure the appropriate and judicious use of antimicrobials. They work together with the clinical team to reduce the unwarranted use of broad-spectrum antibiotics unless indicated (based on clinical and/or lab findings). This enables our unit to minimize the potential of antibiotic resistance, reduce the rate of necrotizing enterocolitis whilst trying to protect the essential but delicate gut flora of the extreme preterm neonate. To further aid this, we routinely start probiotics on all babies admitted under 33 weeks and/or on prolonged broad-spectrum antibiotics.

These twins were both started on standard empiric coverage (ampicillin and gentamicin) antibiotics at birth until their 36-h cultures returned negative. Due to clinical deterioration soon after, we repeated the blood culture and surface swabs. Taking into consideration the maternal history of colonization with an NMD-1 organism sensitive to colistin, colistin was started empirically on the neonates. This is one of the few antimicrobials remaining to which NMD-1 producers are sensitive; however, resistance is rapidly increasing [33]. Other options considered include tigecycline and potentially fosfomycin. Colistin is generally considered a first line therapy (+/- adjunct therapy with a carbapenem), and there is published data regarding both its safety and efficacy in the ELBW population [34,35]. These factors lead to the decision to use colistin as empiric coverage for the CP-CRE. Tigecycline experience is limited on the whole in the pediatric population. The Red Book 2018 states that it should not be used in pediatric patients < six years old due to its adverse effects on tooth enamel, unless no alternative is available [36].

**Figure 4:**
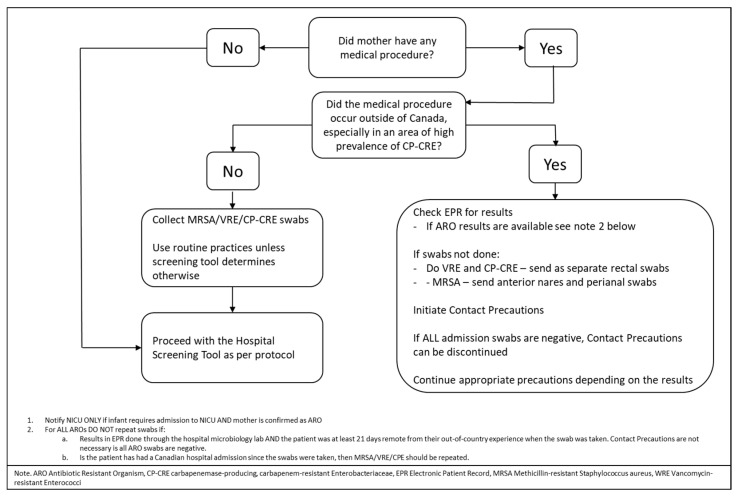
Carbapenemase-producing, carbapenem-resistant Enterobacteriaceae infection and prevention algorithm (for use with the hospital screening tools).

Figure 4 below shows the screening and notification algorithm used for antibiotic-resistant organisms in this case, including CPE. This was an algorithm that included other resistant organisms; it meant that this initial portion could easily be incorporated into existing hospital systems.

Mothers meet the criteria for antibiotic-resistant organism (ARO) screening due to receiving out-of-country hospitalization or care within the last year, as per hospital protocol for the adult population. Patients who received out of country care are high risk for ARO acquisition.

The sensitivity/resistance pattern is monitored in our microbiology lab, on a regular basis for CP-CRE-positive isolates. In our institution, sensitivity testing is performed for every new CP-CRE isolate identified from a rectal screen. Repeat sensitivities are performed on known positives who are re-screened after six months to identify any change in resistance patterns. Additionally, sensitivity testing is conducted for every clinical isolate during the treatment.

Personnel screening was not a consideration. CP-CRE is transmitted via direct and indirect contact. For direct transmission, hand hygiene is the most effective method for disrupting transmission. These babies were placed on contact precautions on admission. CP-CRE has been cultured from hand sink drains. The drains were cultured after infants were discharged on days 0, 7, 14, and 21. Environmental swabs were collected and sent to the lab for culture. All cultures were negative for our cases. The incubator and equipment were disinfected at the beginning of each 12-h shift, and when necessary, with an accelerated hydrogen peroxide disinfectant.

Staff education about CRE infection and spread was vital in implementing and maintaining the policies put in place for the delivery and admissions. Parental education was also important to reassure parents and encourage their active but safe participation in their babies’ care.

**Figure 5:**
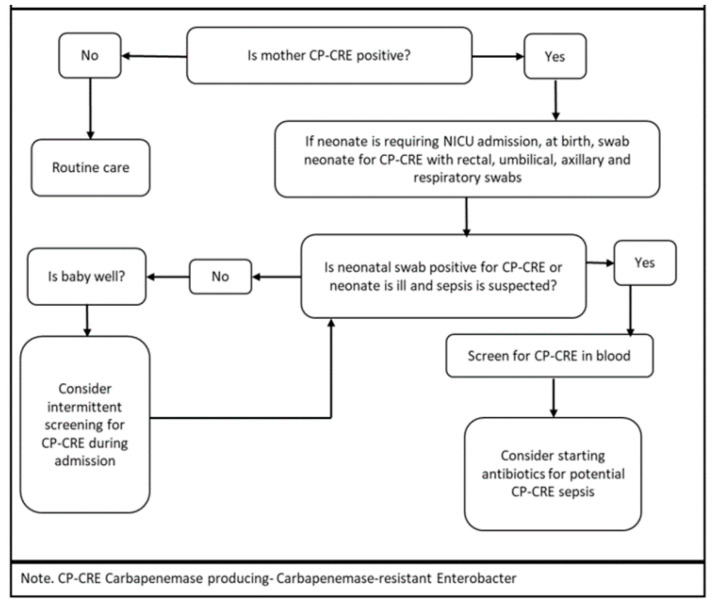
Carbapenemase-producing, carbapenem-resistant Enterobacteriaceae infection and control in neonates admitted to the NICU.

At birth, the management course was primarily dependent on the clinical status of the neonate, as described in Figure 5 below. CP-CRE colonization can be a precursor to infection [14,36,37,38], more so in an immunocompromised population such as neonates. This emphasizes the need for preventive measures and surveillance. Direct contact on the hands of individuals or indirect contact via contaminated equipment result in the transmission of AROs. Newborns eventually become colonized by constant exposure to parents with AROs. Therefore, implementing contact precautions as a pre-emptive strategy for the duration of hospital stay mitigates potential transmission.

At all times during the resuscitation and admission:

- Contact precautions were observed with the medical team using personal protective equipment of gloves and gown.
- Dedicated equipment used with the neonates were kept in their isolation room and cleaned there. The incubators required two-step cleaning when they were changed. It was preferable to change the incubators during the workweek and not weekends to facilitate infection prevention and control inspection of the incubator units prior to and after they were cleaned. Environmental swabs were taken. We left the cleaned incubators (the changed ones) aside until culture results were available. We ensured documentation of the incubator number in the baby’s chart.
- All bodily fluids were secured in their isolation room and disposed of only after being placed in a closed bag.
- Strict hand hygiene was observed for staff and parents with alcohol-based hand rub.
- Breastfeeding was not contraindicated. The mother’s own milk was stored in the refrigerator in the babies’ room. Milk that required fortification (due to prematurity) was sent to the milk preparation room after education of the milk preparation staff for contact precautions as parents would have handled the bottles. Parental bathroom use was restricted to a single bathroom for the entire NICU stay, and no one else was allowed to use this bathroom. This washroom received daily cleaning, and hand sink drains were treated with RESCUE^TM^ disinfectant daily. This disinfectant cleaner is bactericidal to NDM-1 bacteria [39].
- The family did not use the family room as this was a shared space with other families. The family was asked to inform NICU of any development of diarrhea. They were asked to not visit when experiencing diarrhea.
- All relevant staff was aware of the need for the cleaning protocol for equipment and the room once discharged.
- Neonates remained on contact precautions for the duration of their stay due to ongoing exposure.

After discharge from the NICU, the neonates required care in the follow-up clinic. During all in-person clinic visits:

- Appointments were made towards the end of the workday, and so the chance of interaction with and the chance of spread to other families was reduced.
- Once they arrived at the clinic, they were placed immediately in the identified room and not in the waiting area.
- Rooms and equipment were identified specifically for the visit, and these were deep-cleaned with hospital-approved disinfectant. At our institution, we use an accelerated hydrogen-based product. Rooms were cleaned prior to the next day and before the next use.
- Personal protective equipment was used by the staff directly in contact with the family.
- A specified bathroom was identified for their use only and deep-cleaned prior to use again.
- Education of staff as a multidisciplinary team and parents is an essential part of the process for compliance to policies.

### Clinical Implications

The algorithms presented above are based on the evidence from the literature and our clinical experience. We provide this as a potential guide to be used in the setting of maternal colonization of CPE, a high-risk delivery and a neonatal NICU admission. The unique clinical considerations for our population are summarized here in salient points: 

Antenatal period:

- Isolation of parents and bathroom restrictions.
- Screening for CPE colonization, especially if there are identified risk factors as per Public Health Ontario, such as medical procedures or hospital admissions in or outside of Canada during the previous 12 months—for example, invitro fertilization or cervical cerclage.

NICU:

- The decision to treat or not for suspected or confirmed sepsis as the drugs for CP-CRE treatment are infrequently used in neonates. The safety profile, target drug range, length of treatment and dosage in this age group is unknown. An individualized approach to treatment should be considered in this population based on clinical presentation, cultures and sensitivity. The sensitivity of the maternal isolates should also be a factor in the decision process for antibiotic choice.
- Management of infant contact with parents where colonization may occur—for example, attention to hand washing with kangaroo care.
- Management of breastfeeding and human milk expression, handling and storage.
- Limiting exposure to other parents with vulnerable children—no use of shared facilities.

Primary care and neonatal follow up clinic:

- General measures for contact precautions.

## 5. Conclusions

CRE and, by extension, CP-CRE is a present and evolving challenge for neonatal infections. Standardized efforts to curb the acquisition of this infection would be judicious given the challenges of treatment and continued emerging antibiotic resistance in a very susceptible neonatal population. Simple infection control measures can be useful and cost-effective in preventing transmission during the in-hospital admission and in the follow-up in-person visits. Essential to this process is the education of the hospital staff and parents regarding the risk, modes and implications of transmission.

## Figures and Tables

**Table 1:** Risk factors for CPE cases in Ontario, May 2018 to April 2019 (*n* = 289). Clinical aspects related to maternal and newborn care.

Risk Factors	Cases	Proportion (%)	Maternal Scenarios/Considerations	Neonatal Scenarios/Considerations
Chronic illness/underlying medical conditions	241	83.4		
Inpatient hospitalization in Canada in the last 12 months	155	53.6	Admission for obstetrics reasons such as preterm labor, rupture of membranes	Neonates’ risk of admission to the NICU increases, especially with prematurity
Travel outside of Canada in the last 12 months	151	52.2	Travel especially to endemic countries in preconception period or during pregnancy	
Medical/surgical procedure in Canada in the last 12 months	102	35.3	In vitro fertilization, cervical cerclage etcetera	
Healthcare received outside of Canada in last 12 months	92	31.8	Maternal obstetric care in higher-risk countries or contacts from these countries such as India and Pakistan as per Public Health Ontario [14]	
ICU admission in Canada in the last 12 months	55	19.0		Multiple potential reasons in the NICU including respiratory procedures (ventilation, suctioning), breast feeding, or human milk expression and handling
Endoscopic procedure in Canada in the last 12 months	41	14.2		
Previous colonization with CPE	31	10.7	Maternal obstetric care in higher-risk countries or contacts from these countries such as India and Pakistan as per Public Health Ontario [14]	
Known contact with a confirmed case	15	5.2	Maternal obstetric care in higher-risk countries or contacts from these countries such as India and Pakistan as per Public Health Ontario (ref Surveillance report)	Kangaroo care, breast milk expression and breast feeding
Other	84	29.1		

Note. CPE: carbapenem-resistant Enterobacteriaceae; NICU: neonatal intensive care unit. This is adapted from Public Health Ontario [14].

**Table 2:** Published findings of CP-CRE in neonates.

Author	Country	Population	Findings
Zheng et al., 2016	China	NICU admissions in a tertiary care health facility	Eighteen carbapenem-resistant *Klebsiella pneumoniae* strains were isolated from 17 patients in various specimens, including sputum, stool, and blood, and one carbapenem-resistant *Klebsiella pneumoniae* was detected in incubator water.
[20]		Period: in 2014	Nosocomial surveillance systems should play a more important role in the infection control to limit the spread of NDM1-producing pathogens.
Mairi et al., 2019	Algeria	All mothers and their newborns managed in two maternity units	414 mothers and 422 newborns were included.
			836 rectal swabs and 221 vaginal swabs were collected.
[21]		Period: January 2016 to April 2016	A total of 28 CPE isolates were obtained from mothers (*n* = 19) (with two different isolated from the same mother), newborns (*n* = 7), and the environment (*n* = 1).
			Low birth weight was significantly associated with CPE carriage in the newborn.
			No carriers were premature, suggesting that CPE had no direct role on a premature delivery and could not represent a high risk for infections encountered frequently in preterm newborns but could influence birth weight.
			Focused interventions to reduce this cross-transmission in settings of high endemicity are required.
Arhoune et al., 2017	Morocco	All neonates hospitalized in the NICU	A high rate of multi-resistance ESBLE was noted. The prevalence of carbapenemase-producing Enterobacteriaceae was 1.8%. It was proposed a screening policy be developed
[22]		Period: February 2013 to July 2013	
Jimenez Ramila et al., 2018	Spain	All pregnant women at the time of delivery and their newborns	A total of 815 women and 800 neonates were studied; 59 were found to be colonized by ESBLE at delivery (prevalence, 7.2%; 95% CI: 5.6–9.2) but no CPE were found.
[23]		Period: August 2014 to June 2015	
Mougkou et al., 2012 [24]	Greece	Multi-center, eight NICUs in five public hospitals.	Rectal and umbilical swabs from infants in 8 NICUs.
(Abstract)		Period: November 2011 to January 2012.	One outborn neonate was colonized. The study showed a very low prevalence of colonization with CPE in NICU patients. Factors associated with this phenomenon need to be further determined.
Singh et al., 2018	India	Three rectal swabs were collected from hospital-delivered and NICU-admitted neonates	From 300 neonates, 26 cases of CRE were isolated.
			Statistically, significant risk factors were NG tube, breastfeeding, NG feeding, top feeding, expressed breastmilk, ventilation, antibiotic administration, and hospitalization duration.
[25]			Top feeding and antibiotics administration were identified as two independent risk factors by multiple logistic regression.
			Active surveillance of cultures from hospitalized patients and implementation of preventive efforts can reduce the risk of CRE.
Ballot et al., 2019	South Africa	A cross-sectional retrospective review of multidrug-resistant organisms in neonates admitted to a tertiary neonatal unit.	A total of 465 infections in 291 neonates. Very low birth weight was record in 68.6% of cases.
[26]		Period:1 January 2013 to December 2015.	The median age of infection was 14.0 days. Risk factors for MDRE included prematurity (*p* = 0.01), lower birth weight (*p* = 0.04), maternal HIV infection (*p* = 0.02) and oxygen on day 28 (*p* < 0.001).
			There was an increase in CRE from 2.6% in 2013 to 8.9% in 2015 (*p* = 0.06).
			There was an increase in CRE from 2.6% in 2013 to 8.9% in 2015 (*p* = 0.06). Most of the CRE were NDM producers.

Note. CPE: carbapenemase-producing Enterobacter; CRE: carbapenem-resistant Enterobacteriaceae; ESBLE: extended-spectrum b-lactamase-producing Enterobacteriaceae; HIV: human immunodeficiency virus; MDRE: multidrug-resistant Enterobacteriaceae; NICU: neonatal intensive care unit; NDM: New Delhi metallo—β lactamase.

## Supplementary Materials

The following are available online at https://www.mdpi.com/article/10.3390/children8050399/s1, Table S1: Summary of microbiology cultures.

## References

[1] Cailes B., Kortsalioudaki C., Buttery J., Pattnayak S., Greenough A., Matthes J., Russell A.B., Kennea N., Heath P.T. (2018). Epidemiology of UK neonatal infections: The neonIN infection surveillance network. Arch. Dis. Child. Fetal Neonatal Ed..

[2] Potter R.F., D’Souza A.W., Dantas G. (2016). The rapid spread of carbapenem-resistant Enterobacteriaceae. Drug Resist. Updat..

[3] Laxminarayan R., Duse A., Wattal C., Zaidi A.K.M., Wertheim H.F.L., Sumpradit N., Vlieghe E., Hare G.L., Gould I.M., Goossens H. (2013). Antibiotic resistance—the need for global solutions. Lancet Infect Dis..

[4] Tsai M.-H., Chu S.-M., Hsu J.-F., Lien R., Huang H.-R., Chiang M.-C., Fu R.-H., Lee C.-W., Huang Y.-C. (2014). Risk Factors and Outcomes for Multidrug-Resistant Gram-Negative Bacteremia in the NICU. Pediatrics.

[5] Paterson D.L., Doi Y. (2007). A Step Closer to Extreme Drug Resistance (XDR) in Gram-Negative Bacilli. Clin. Infect. Dis..

[6] Guidance for Control of Infections with Carbapenem-Resistant or Car-Bapenemase-Producing Enterobacteriaceae in Acute Care Facilities. https://www-cdc-gov.myaccess.library.utoronto.ca/mmwr/preview/mmwrhtml/mm5810a4.htm.

[7] Righi E., Peri A.M., Harris P.N.A., Wailan A.M., Liborio M., Lane S.W., Paterson D.L. (2016). Global prevalence of carbapenem resistance in neutropenic patients and association with mortality and carbapenem use: Systematic review and meta-analysis. J. Antimicrob. Chemother..

[8] Durante-Mangoni E., Andini R., Zampino R. (2019). Management of carbapenem-resistant Enterobacteriaceae infections. Clin. Microbiol. Infect..

[9] Public Health Ontario Ontario Agency for Health Protection and Promotion. https://www.publichealthontario.ca/en/health-topics/infection-prevention-control/routine-practices-additional-precautions.

[10] Carbapenemases in Enterobacteriaceae: Detection and Antimicrobial Therapy. https://www.frontiersin.org/article/10.3389/fmicb.2019.01823.

[11] Infection Control CDC Reports that CRE Are a Nightmare Bacteria in Healthcare Environments. Dow Jones Factiva, Life Science Weekly. https://global-factiva-com.myaccess.library.utoronto.ca/redir/default.aspx?P=sa&NS=16&AID=9UNI011000&an=LFSW000020130322e93q0011t&cat=a&ep=ASI.

[12] Mataseje L.F., Abdesselam K., Vachon J., Mitchel R., Bryce E., Roscoe D., Boyd D.A., Embree J., Katz K., Kibsey P. (2016). Results from the Canadian Nosocomial Infection Surveillance Program on Carbapenemase-ProducingEnterobacteriaceae, 2010 to 2014. Antimicrob. Agents Chemother..

[13] Public Health Ontario Carbapenemase-Producing Enterobacteriaceae Frequently Asked Questions. https://www.publichealthontario.ca/-/media/documents/F/2019/faq-cpe.pdf?la=en.

[14] Surveillance Report: CPE in Ontario, 1 May 2018–30 April 2019. https://www.publichealthontario.ca/-/media/documents/surveillance-reports/cpe/surveillance-report-cpe-2019.pdf?la=en.

[15] De Geyter D., Blommaert L., Verbraeken N., Sevenois M., Huyghens L., Martini H., Covens L., Piérard D., Wybo I. (2017). The sink as a potential source of transmission of carbapenemase-producing Enterobacteriaceae in the intensive care unit. Antimicrob. Resist. Infect. Control..

[16] Tang L., Tadros M., Matukas L., Taggart L., Muller M. (2020). Sink and Drain Monitoring and Decontamination Protocol for Carbapenemase-producing Enterobacteriaceae (CPE). Am. J. Infect. Control..

[17] Kohler P.P., Melano R.G., Patel S.N., Shafinaz S., Faheem A., Coleman B.L., Green K., Armstrong I., Almohri H., Borgia S. (2018). Emergence of Carbapenemase-Producing Enterobacteriaceae, South-Central Ontario, Canada1. Emerg. Infect. Dis..

[18] (2016). Centers for Disease Control and Prevention, National Center for Emerging and Zoonotic Infectious Diseases (NCEZID), Divi-sion of Healthcare Quality Promotion (DHQP) [Internet]. https://www-cdc-gov.myaccess.library.utoronto.ca/infectioncontrol/basics/transmission-based-precautions.html.

[19] Peleg A.Y., Franklin C., Bell J.M., Spelman D.W. (2005). Dissemination of the Metallo—Lactamase Gene blaIMP-4 among Gram-Negative Pathogens in a Clinical Setting in Australia. Clin. Infect. Dis..

[20] Zheng R., Zhang Q., Guo Y., Feng Y., Liu L., Zhang A., Zhao Y., Yang X., Xia X. (2016). Outbreak of plasmid-mediated NDM-1-producing Klebsiella pneumoniae ST105 among neonatal patients in Yunnan, China. Ann. Clin. Microbiol. Antimicrob..

[21] Mairi A., Touati A., Bessai S.A., Boutabtoub Y., Khelifi F., Sotto A., Lavigne J.-P., Pantel A. (2019). Carbapenemase-producing Enterobacteriaceae among pregnant women and newborns in Algeria: Prevalence, molecular characterization, maternal-neonatal transmission, and risk factors for carriage. Am. J. Infect. Control..

[22] Arhoune B., Oumokhtar B., Hmami F., Barguigua A., Timinouni M., El Fakir S., Bouharrou A. (2017). Rectal carriage of extended-spectrum β-lactamase- and carbapenemase-producing Enterobacteriaceae among hospitalised neonates in a neonatal intensive care unit in Fez, Morocco. J. Glob. Antimicrob. Resist..

[23] Jiménez-Rámila C., López-Cerero L., Martín M.V.A., Martín C.V., Serrano L., Pascual Á., Rodríguez-Baño J. (2019). Vagino-rectal colonization and maternal–neonatal transmission of Enterobacteriaceae producing extended-spectrum β-lactamases or carbapenemases: A cross-sectional study. J. Hosp. Infect..

[24] Mougkou K., Michos A., Spyridopoulou K., Daikos G.L., Siahanidou T., Spyridis N., Kapetanakis J., Korkas A., Anagnostakou C., Papagaroufalis G. Colonization of neonates with carbapenemase-producing Enterobacteriaceae (CPE). Proceedings of the 30th Annual Meeting of the European Society for Paediatric Infectious Diseases (ESPID 2012).

[25] Singh N.P., Das Choudhury D., Gupta K., Rai S., Batra P., Manchanda V., Saha R., Kaur I. (2018). Predictors for gut colonization of carbapenem-resistant Enterobacteriaceae in neonates in a neonatal intensive care unit. Am. J. Infect. Control..

[26] Ballot D.E., Bandini R., Nana T., Bosman N., Thomas T., Davies V.A., Cooper P.A., Mer M., Lipman J. (2019). A review of -multidrug-resistant Enterobacteriaceae in a neonatal unit in Johannesburg, South Africa. BMC Pediatr..

[27] Magiorakos A.P., Burns K., Baño J.R., Borg M., Daikos G., Dumpis U., Lucet J.C., Moro M.L., Tacconelli E., Simonsen G.S. (2017). Infection prevention and control measures and tools for the prevention of entry of carbapenem-resistant Enterobacteriaceae into healthcare settings: Guidance from the European Centre for Disease Prevention and Control. Antimicrob. Resist. Infect. Control..

[28] Schneider A., Coope C., Michie S., Puleston R., Hopkins S., Oliver I. (2019). Implementing a toolkit for the prevention, management and control of carbapenemase-producing Enterobacteriaceae in English acute hospitals trusts: A qualitative evaluation. BMC Health Serv. Res..

[29] Fournier S., Monteil C., Lepainteur M., Richard C., Brun-Buisson C., Jarlier V., Collective AP-HP Outbreaks Control Group (2014). Long-term control of carbapenemase-producing Enterobacteriaceae at the scale of a large French multihospital institution: A nine-year experience, France, 2004 to 2012. Eurosurveillance.

[30] Chair M.R., Cruickshank M., Cheng A., Gandossi S., Quoyle C., Stuart R., Sutton B., Turnidge J., Bennett N., Buising K. (2017). Recommendations for the control of carbapenemase-producing Enterobacteriaceae (CPE): A guide for acute care health facilities. Infect. Dis. Health.

[31] Jamal A.J., Garcia-Jeldes F., Baqi M., Borgia S., Johnstone J., Katz K., Kohler P., Muller M.P., McGeer A.J., for the CPE Investigators of the Toronto Invasive Bacterial Diseases Network (2019). Infection prevention and control practices related to carbapenemase-producing Enterobacteriaceae (CPE) in acute-care hospitals in Ontario, Canada. Infect. Control. Hosp. Epidemiol..

[32] Castagnola E., Tatarelli P., Mesini A., Baldelli I., La Masa D., Biassoni R., Bandettini R. (2019). Epidemiology of carbapenemase-producing Enterobacteriaceae in a pediatric hospital in a country with high endemicity. J. Infect. Public Health.

[33] Wangchinda W., Pati N., Maknakhon N., Seenama C., Tiengrim S., Thamlikitkul V. (2018). Collateral damage of using Colistin in hos-pitalized patients on emergence of colistin-resistant Escherichia coli and Klebsiella pneumoniae colonization and infection. Antimicrob. Resist. Infect. Control.

[34] Jajoo M., Kumar V., Jain M., Kumari S., Manchanda V. (2011). Intravenous Colistin Administration in Neonates. Pediatr. Infect. Dis. J..

[35] Çağan E., Baş E.K., Asker H.S. (2017). Use of Colistin in a Neonatal Intensive Care Unit: A Cohort Study of 65 Patients. Med Sci. Monit..

[36] Kimberlin D.W. (2018). Red Book: 2018–2021 Report of The Committee on Infectious Diseases.

[37] Borer A., Saidel-Odes L., Eskira S., Nativ R., Riesenberg K., Livshiz-Riven I., Schlaeffer F., Sherf M., Peled N. (2012). Risk factors for developing clinical infection with carbapenem-resistant Klebsiella pneumoniae in hospital patients initially only colonized with carbapenem-resistant K pneumoniae. Am. J. Infect. Control..

[38] Schechner V., Kotlovsky T., Kazma M., Mishali H., Schwartz D., Navon-Venezia S., Schwaber M., Carmeli Y. (2013). Asymptomatic rectal carriage of blaKPC producing carbapenem-resistant Enterobacteriaceae: Who is prone to become clinically infected?. Clin. Microbiol. Infect..

[39] Virox Technologies Incorporated RescueTM Ready to Use One Step Disinfectant Cleaner & Deodorizer. [Internet]. https://rescuedisinfectants.com/wp-content/uploads/2017/07/REF067_Rescue-RTU-Ref-Sheet_17-069-2.pdf.

