# Supplementary material for: Neonates with Maternal Colonization of Carbapenemase-Producing, Carbapenem-Resistant Enterobacteriaceae: A Mini-Review and a Suggested Guide for Preventing Neonatal Infection
Source: Children (Basel). 2021 May 15;8(5):399. doi: 10.3390/children8050399 (PMC8156425; doi:10.3390/children8050399)
Supplement: Supplementary file 1 [file children-08-00399-s001.zip › children-1155336-supplementary.pdf]

CP-CRE Mini-review

Supplementary Material

Table S1. Summary of microbiology cultures.

Day of life	Clinical indicator	Blood culture	Urine culture	CSF culture	Swabs done	Sensitivity pattern		Antibiotics administered
						Antibiotic	Sensitivity	
**Case 1 Twin A**								
**1**	Cardiorespiratory distress at birth, premature spontaneous labor	No growth after 36 hours incubation			Rectal - Klebsiella pneumoniae (ESBL producer) – heavy growth<br>Axilla - Klebsiella pneumoniae (ESBL producer) – light growth.<br>ETT - No Ureaplasma urealyticum isolated.<br>No Large colony of Mycoplasma isolated	Klebsiella pneumoniae<br>Ampicillin<br>Cefotaxime/<br>Cefotolozane Tazobactam<br>Gentamicin<br>Meropenem<br>Tigecycline	 R<br>R<br>S<br>S<br>S<br>S	Empiric ampicillin and gentamicin commenced at birth. Gentamicin switched to Colistin with further clinical deterioration. Colistin given as intravenous 5mg/kg/day divided q 8 hourly. Antibiotics stopped with negative blood culture result at 36 hours
**3**	Surveillance				ETT, umbilical, rectal for NDM1 E. coli - No			

					carbapenemase-producing organisms isolated.			
**8**	Surveillance				ETT, umbilical, rectal, axillary for NDM1 CPE - No growth			
**29**	Persistent tachycardia HR 190-200, lethargy, C-reactive protein increasing from 68 to 88	No growth after 36 hours incubation	Escherichia coli (non-ESBL producing strain)	No growth		**Escherichia coli**<br>Ampicillin<br>Cefotaxime<br>Gentamicin<br>Meropenem	 S<br>S<br>S<br>S	Empiric ampicillin, Colistin and Meropenem commenced. Ampicillin only continued for 7 days with culture results
**30**	Surveillance				ETT, umbilical, rectal, axillary for the carbapenemase-producing organism - No growth			
**43**	Surveillance				Nose and perianal/rectal - No growth			

Case 1 Twin B

**1**	Cardiorespiratory distress at birth, premature spontaneous labour	No growth after 36 hours incubation			Rectal - Klebsiella pneumoniae (ESBL producer) – light growth. Axilla, Gastric aspirate - No growth	**Klebsiella pneumoniae**<br>Ampicillin<br>cefotaxime<br>/<br>Cefotolozane<br>tazobactam<br>gentamicin<br>Meropenem	 R<br>R<br><br>S<br>S<br>S<br>S	Empiric ampicillin and gentamicin commenced at birth. Gentamicin switched to Colistin with further clinical

					Tigecycline		deterioration. Colistin given as intravenous 5mg/kg/day divided q 8 hourly. Antibiotics stopped with negative blood culture result at 36 hours
**3**	Surveillance			ETT, umbilical, rectal for NDM1 E. coli - No carbapenemase-producing organisms isolated			
**8**	Surveillance			ETT, umbilical, rectal, axillary for NDM1 CPE - No growth			
**15**	Blood in stool, necrotizing enterocolitis considered but ruled out	No growth after 36 hours incubation					No antibiotics commenced
**30**	Surveillance			ETT, umbilical, rectal, axillary for the carbapenemase-producing organism – No growth			
**37**	Increasing ventilatory requirement, right upper lobe collapse chest	No growth after 36 hours incubation	No growth	ETT - Heavy growth of Staphylococcus aureus, moderate growth of	**Staphylococcus aureus**<br>Cloxacillin<br>Penicillin<br>Vancomycin<br>**Klebsiella pneumoniae**	 S<br>R<br>S	

	radiograph, initially improved with ventilatory maneuvers				Klebsiella pneumoniae (ESBL producer) and scant growth of Escherichia coli	Ampicillin<br>Cefotaxime<br>Gentamicin<br>Meropenem<br>**Escherichia coli**<br>Ampicillin<br>Cefotaxime<br>Gentamicin<br>Meropenem	**R**<br>**R**<br>**S**<br>**S**<br><br>**S**<br>**S**<br>**S**<br>**S**	
**42**	Persisting increasing ventilatory requirement				ETT aspirate - Staphylococcus aureus and Escherichia coli (ESBL producer)	**Staphylococcus aureus**<br>Cloxacillin<br>Penicillin<br>Vancomycin<br>**Escherichia coli**<br>Ampicillin<br>Cefotaxime<br>Gentamicin<br>Meropenem	 S<br>R<br>S<br><br>**R**<br>**R**<br>**S**<br>**S**	Ertapenem for 10 days for ventilator acquired pneumonia
**43**	Increasing oxygen requirement, related to symptomatic patent ductus arteriosus	No growth after 36 hours incubation	1 to 10 X E5 CFU/L Coagulase-negative staphylococci			**Coagulase-negative staphylococci**<br>Ampicillin<br>Cloxacillin<br>Vancomycin	 **R**<br>**R**<br>**S**	Assumed contaminant
**63**	Surveillance				Umbilical, nose and perianal/rectal - No growth			

Note. CPE= carbapenemase-producing Enterobacteriaceae, CFU= colony forming units, CSF= cerebrospinal fluid, E.coli = Escherichia coli, ESBL= extended spectrum beta-lactamases, ETT=endotracheal tube, NDM= New Delhi metallo-ß-lactamase, PCR= polymerase chain reaction, R= resistant, S = sensitive.
